# Modelling the cost-effectiveness of HIV care shows a clear benefit when transmission risk is considered in the calculations – A message for Central and Eastern Europe

**DOI:** 10.1371/journal.pone.0186131

**Published:** 2017-11-13

**Authors:** Justyna D. Kowalska, Grzegorz Wójcik, Jakub Rutkowski, Magdalena Ankiersztejn-Bartczak, Ewa Siewaszewicz

**Affiliations:** 1 Department of Adults’ Infectious Diseases, Medical University of Warsaw, Warsaw, Poland; 2 HIV Out-Patients Clinic, Hospital for Infectious Diseases in Warsaw, Warsaw, Poland; 3 HTA Consulting, Cracow, Poland; 4 Foundation of Social Education (FES), Warsaw, Poland; 5 Gilead Sciences, Warsaw, Poland; University of New South Wales, AUSTRALIA

## Abstract

**Background:**

HIV epidemic remains a major global health issue. Data from cost-effectiveness analyses base on CD4+ count and morbidity in patients with symptomatic and asymptomatic HIV infection. The approach adopted in these analyses includes many other factors, previously not investigated. Additionally, we evaluate the impact of sexual HIV transmission due to delayed cART on the cost-effectiveness of care.

**Methods:**

A lifetime Markov model (1-month cycle) was developed to estimate the cost per quality adjusted life years (QALY) for a 1- and 3-year delay in starting cART (as compared to starting immediately at linkage to care) lifetime costs, clinical outcomes and cost-effectiveness. Patients were categorized into having asymptomatic HIV, AIDS, Hodgkin’s Lymphoma, and non-AIDS defining condition. Mortality rates and utility values were obtained from published literature. The number of new infected persons was estimated on the basis of sexual orientation, the number of sexual partners per year, the number of sex acts per month, frequency of condom use and use of cART. For the input Test and Keep in Care (TAK) project cohort data were used. Costs of care, cART and potential life-years lost were based on estimated total costs and the difference in expected QALY gained between an HIV-positive and an average person in Polish population. Costs were based on real expenditures of the Ministry of Health, National Health Fund, available studies and experts’ opinion. Costs and effects were discounted at rates of 5% and 3.5%, respectively.

**Results:**

Input data were available for 141 patients form TAK cohort. The estimated number of new HIV infections in low, medium and high risk transmission groups were 0.28, 0.61, 2.07 with 1 and 0.82, 1.80, 6.11 with a 3-year delay, respectively. This reflected QALY loss due to cART delay of 0.52, 1.13, 3.84 and 2.02, 4.43, 15.03 for a 1- and 3-year delay, respectively. If additional costs of treatment and potential life-years lost due to new HIV infections were not taken into account, initiating cART immediately at linkage to care was not cost-saving irrespective of cART delay. Otherwise, when additional costs and QALY lost due to new HIV infections were included, immediate cART initiation was cost-saving regardless of the chosen scenarios.

**Conclusions:**

If new HIV infections are not taken into account, then starting cART immediately does not dominate comparing to delaying cART. When taking into account HIV transmission in cost–effectiveness analysis, immediate initiation of HIV treatment is a profitable decision from the public payer’s perspective.

## Introduction

HIV epidemic remains one of the most challenging areas in both global and national health management. The accomplishments of the recent 15 years have brought less toxic and highly effective antiretroviral therapy, leading to a significant decrease in both morbidity and mortality of HIV positive persons [1, 2]. However lives improved and lives saved do not always translate into economic savings, due to high costs of treatment and care, the life-long character of such care and continuously increasing number of people to treat [3].

In comparison with the number of already diagnosed persons, the number of new infections is substantial and remains quite stable every year. According to the most recent UNAIDS report published in 2016, the number of people living with HIV increased globally from 33.3 million in 2010 to 36.7 million in 2015 with the number of people on combination antiretroviral therapy (cART) increasing from 7.5 million to over 17 million [4]. The gains in treatment are to be responsible for a 26% decrease in deaths, which in turn translates into more people requiring treatment and care, namely more costs.

At the same time recent years have brought strong evidence for the role of cART in protecting HIV transmission through sexual acts when an HIV positive person is on effective treatment [5, 6]. It is, therefore, of great interest for both health care providers and public payers to understand the economic impact of investing in early cART on the savings made through prevention of HIV transmission [7].

For national strategies which would prioritise early cART among other life-saving therapies, governments and stakeholders require tools that are developed on the basis of national or regional epidemiology, the costs and health structure. Cost-effectiveness analyses have recently become widely accepted as the standard approach to health. In our model we applied, as advised by the World Health Organization, the convention to report the intervention as “cost-saving” (both cheaper and better) with the absolute magnitude of savings and health gains [8].

Finally we have used real life data from the existing cohort of HIV positive persons followed in the Test and Keep in Care (TAK) project. The TAK project collects information on patients diagnosed with HIV between 2010 and 2013 in voluntary counselling and testing centres (VCTs) in central Poland, follows their linkage to HIV clinics and further routine clinical care. A unique characteristic of this cohort is the availability of information provided by VCTs clients in a standard anonymous questionnaire before performing an HIV test, including detailed information about sexual behaviors and preferences [9, 10].

## Methods

**Fig 1:**
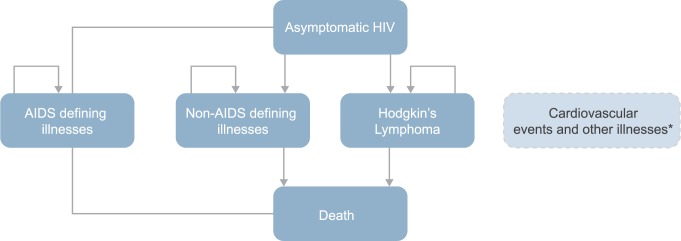
Markov model for HIV treatment. *They do not determine independent health states. Only additionally costs and deaths due to cardiovascular events and other illnesses were charged, regardless of the state of health in each cycle of analysis.

A lifetime Markov model was built to estimate quality adjusted life years (QALY) and costs of treatment of an HIV infected patient, from the public payer’s perspective (Fig 1). The model was developed in Microsoft Excel 2013 with Visual Basic Applications. The model allowed to perform cost-utility analysis, to estimate the ratio of incremental cost per additional year of life in full health (ICER) and to perform probabilistic sensitivity analysis, in order to allow the estimation of confidence intervals for deterministic results and scatter of results. In addition, a cost-effectiveness acceptability curve (CEAC) was generated to test for the effectiveness threshold.

Our analyses were performed to compare two hypothetical, independent cohorts of patients, one linked to immediate care and cART treatment (Immediate Initiation Group, IIG), while the second cohort started cART treatment after an arbitrarily chosen time delay (Deferred Initiation Group, DIG). Modelling the distribution of patients in these two cohorts took place in two independent steps. First, based on the baseline characteristics of patients followed in TAK cohort: mean CD4+ cells count, median HIV RNA and others (Table 1), an average level of CD4+ cells count was estimated for both cohorts in each cycle of the predetermined time horizon (S1 File). Next, using predetermined input parameters and specified CD4+ cell count, the main Markov model simulation was performed.

**Table 1:** Baseline characteristic of patients from TAK cohort.

Parameters	Value
**Mean CD4+ count [cells/mm3]**	413.25
**Median HIV RNA [copies/ml]**	28 414
**Mean age [years]**	36
**Females pct. [%]**	6.00
**Black Race pct. [%}**	0.00
**Transmission by IDU [%]**	1.00
**Heterosexual pct. [%]**	9.93
**Homosexual pct. [%]**	**83.69**
**Bisexual pct. [%]**	**5.67**

The model has one-month cycles and takes into account 33 events or illnesses divided into 18 health states and 8 additional events or diseases affecting estimated costs and the length of life (Table 2). The baseline state of the model is an asymptomatic HIV, that was the people with HIV who did not experience additional comorbidities. In each cycle of analysis, patients were distributed between health states with assigned corresponding probabilities. We made the assumption, that after changing baseline health state it was not possible for the person to change their health state, except for death incidence, there was no possibility of the occurrence of the same event repeatedly and no possibility of having several diseases at the same time (Fig 1). In other words, health states representing AIDS-defining illnesses, non-AIDS defining illnesses, and Hodgkin's Lymphoma were transient states between an asymptomatic HIV and death. Due to insufficient data on conditional transitions, a simplified assumption was adopted. This approach in case of the cohort of patients allows to obtain results corresponding to reality.

**Table 2:** Illnesses and events included in analysis.

Illnesses / event	Category		Info
**Asympthomatic HIV**			Health state
**Non-Hodgkin’s lymphoma**	Severe	AIDS-definig illnesses	Health state
**Progressive multifocal leukoencephalopathy**			Health state
**Cryptococcosis**	Moderate		Health state
**Cerebral toxoplasmosis**			Health state
**Invasive cervical carcinoma**			Health state
**Bacterial pneumonia**			Health state
**AIDS dementia complex**			Health state
**Disseminated Mycobacterium avium disease**			Health state
**HIV wasting syndrome**	Mild		Health state
**Pulmonary tuberculosis**			
**Pneumocystis jiroveci (carinii) pneumonia**			
**Extrapulmonary tuberculosis**			
**Esophageal candidiasis**			
**Cryptosporidiosis**			
**Cytomegalovirus infection**			
**Kaposi sarcoma**			
**Herpes simplex disease**			
**Liver cancer**	Non-AIDS defining illnesses		Health state
**Lung cancer**			Health state
**Anal cancer**			Health state
**Pancreatitis**			Health state
**End-stage renal disease**			Health state
**Liver associated event**			Health state
**Hodgkin's lymphoma**	Hodgkin's lymphoma		Health state
**Myocardial infarction: fatal**	Cardiovascular events and other illnesses		Additional event
**Myocardial infarction: nonfatal**			Additional event
**Stroke: fatal**			Additional event
**Stroke: nonfatal**			Additional event
**Invasive procedures (coronary artery coronary by-pass, carotid endarterectomy)**			Additional event
**Deaths from other CHD**			Additional event
**Fracture, inadequate trauma**			Additional disease
**Diabetes mellitus**			Additional disease
**Death**			Health state

In addition, based on presumed sexual behaviour of a person, the proportion of new HIV-infected people through sexual contact was estimated. New infections were calculated for a set period of time equal to the treatment delay in the base case scenario. Moreover, the costs and life years lost for newly infected persons were calculated (detailed description in “Transmission Model” part).

### CD4+ model

The prediction of mean level of CD4+ cell count was independent for each modelled cohort. In both IIG and DIG cohort we assumed the average level of CD4+ lymphocytes caused by treatment according to the data published by Mocroft et al. in 2007[11]. Additionally, we have assumed that once a patient achieved full treatment response (serum HIV RNA<50 copies/ml), it was maintained throughout the analysed period of time. However, a decline in the mean CD4+ cell count until the initiation of cART treatment was programmed for DIG cohort based on the linear model presented by the Natural History Project Working Group for the Collaboration of Observational HIV Epidemiological Research Europe (COHERE) in EuroCoord [12].

### Disease progression probabilities

Specific transition probabilities depend on the current age or previously estimated mean lymphocyte CD4+ count in each model cycle. In case of AIDS-defining illnesses, we used data about the frequency of various diseases related to different levels of CD4+ cells count, retrieved from Mocroft 2013 [13]. Incidence rates of AIDS-defining illnesses per 1000 patients were converted into monthly probabilities of the occurrence of illness. For other non-AIDS related diseases and events, probabilities were linked in each model cycle with the actual age, grouped into 3 categories: <50, 50–64 and at least 65 years, respectively, based on published data [14–18]. In addition, we assumed that regardless of being in one of defined health states, the analyzed patient can independently experience fatal and nonfatal cardiovascular event. We used the probability of cardiovascular events as depicted by Friis-Moller et al. 2015 [17]. The probability values in each of three age-related categories were modified by the adequate risk ratio published by Petoumenos 2014 [19]. We have also assumed that the occurrence of a cardiovascular event did not affect the quality of life and generates only additional costs of events and deaths. As part of the searches performed, no data was found about decrease in QOL among HIV infected patients after a cardiovascular event. In addition, the structure of the model (cardiovascular events are not separate states of health, but only events) makes it difficult to implement possible values. Also, it should be noted, that the probability of cardiovascular event is the same regardless of the type of cART used. The above simplified assumption is referred to as conservative approach (we charge more costs in the DIG arm than in the IIG). The summary of risk of illnesses used in the model is presented in Tables 3 and 4.

**Table 3:** Estimated monthly probability of occurence of AIDS defining illnesses according current CD4+ cells count.

Illnesses	Current CD4+ cells count					
	0–199^a^	200–349	350–499	500–749	750–999	- ≥1000
**Non-Hodgkin’s lymphoma**	0.0175%	0,0175%	0.0089%	0.0055%	0.0031%	0.0040%
**Progressive multifocal leukoencephalopathy**	0.0051%	0.0051%	0.0020%	0.0012%	0.0005%	0.0005%
**Cryptococcosis**	0.0012%	0.0012%	0.0006%	0.0005%	0.0001%	0.0001%
**Cerebral toxoplasmosis**	0.0051%	0.0051%	0.0051%	0.0019%	0.0010%	0.0005%
**Invasive cervical carcinoma**	0.0005%	0.0005%	0.0003%	0.0002%	0.0002%	0.0002%
**Bacterial pneumonia**	0.0117%	0.0117%	0.0068%	0.0041%	0.0035%	0.0023%
**AIDS dementia complex**	0,0096%	0.0096%	0.0050%	0.0039%	0.0034%	0.0023%
**Disseminated Mycobacterium avium disease**	0.0024%	0.0024%	0.0006%	0.0004%	0.0005%	0.0003%
**Mild AIDS^b^**	0.1147%	0.1147%	0.0557%	0.0335%	0.0248%	0.0217%

a) In the study Mocroft 2013, the probability of the mentioned diseases were stratified for CD4+ level greater than 200. We have assumed that the probability in the range 0–199 is the same as for 200–349.

b) Mild AIDS is defined based on Mocroft 2009 [PMID: 19275498]. We included disseminated mycobacterium avium disease, pulmonary tuberculosis, pneumocystis jiroveci pneumonia, extrapulmonary tuberculosis, esophageal candidiasis, cryptosporidiosis, cytomegalovirus infection, Kaposi sarcoma, Herpes simplex disease.

**Table 4:** Estimated monthly probability of occurrence of others illnesses or events included in analysis according current age of patients.

Illnesses / event	Mean	Age <50	Age 50–64	Age> = 65	Source
**Hodgkin's lymphoma**					
**Hodgkin's lymphoma**	0.004%				Worm 2013 [16]
** Non-AIDS defining illnesses**					
**Liver cancer**	0.003%				Worm 2013
**Lung cancer**	0.007%				Worm 2013
**Anal cancer**	0.004%				Worm 2013
**Pancreatitis**	0.010%	0.007%	0.019%	0.014%	Hasse 2011 [18]
**End-stage renal disease**	0.001%				Ryom 2014 [14]
**Liver associated event**	0.021%	0.019%	0.030%	0.007%	Hasse 2011
**Cardiovascular events and other diseases**					
**Fatal myocardial infraction**	0.0036%	0.0031%	0.0067%	0.0090%	Friis-Moller 2015 [17]
**Fatal stroke**	0.0018%	0.0015%	0.0039%	0.0057%	Friis-Moller 2015
**Fatal CHD^a^**	0.0020%	0.0017%	0.0042%	0.0062%	Friis-Moller 2015
**Non-fatal myocardial infraction**	0.0185%	0.0162%	0.0345%	0.0464%	Friis-Moller 2015
**Non-fatal stroke**	0.0114%	0.0098%	0.0246%	0.0364%	Friis-Moller 2015
**Invasive procedure**	0.0080%	0.0069%	0.0168%	0.0246%	Friis-Moller 2015
**Fracture**	0.0137%	0.0065%	0.0263%	0.0426%	Hasse 2011
**Diabetes Mellitus**	0.0352%	0.0352%	0.0352%	0.0352%	Petoumenos 2012 [15]

a) Coronary Heart Disease (CHD)

### Transmission model

In our model the HIV transmission was assumed to occur only through sexual contacts. The probability of infection through the sex act was conditional on the type of sexual intercourse, as published by Lasry et al. in 2014 [20]. In addition to raw probabilities of transmission per one sexual act we have included the factor of condom use and cART treatment. We made an assumption, that the only factor determining the rate of HIV transmission between groups is the presence of cART therapy, thus the overall difference in HIV transmission between IIG and DIG persons stems from the difference in transmission rate due to presence or absence of cART and the time lag to cART therapy initiation (in our analysis it was a 1- or 3-year delay). The input data for sexual orientation, the average number of sexual partners and sexual acts used in the transmission model were retrieved from Test and Keep in Care project (Table 1) [10]. Three sexual orientations were included in the model (homosexual, heterosexual and bisexual) and we assumed that heterosexual persons had only vaginal intercourse, homosexual have insertive (50%) and receptive (50%) anal sex, and bisexuals had each of them (vaginal, insertive and receptive anal sex in equal part). Additionally, we assumed that each patient from the analysed cohort had the same number of intercourses and had the same number of intercourses with each sexual partner.

We have arbitrarily chosen three profiles of the risk of transmission. For the medium risk scenario, which was considered a baseline model, the rate of transmission was estimated assuming that an average HIV positive person has 10 partners per year, 10 monthly sex acts and 50% frequency of condom use per act. For the low and high risk scenarios used in sensitivity analyses, we assumed a person to have 3 and 50 partners per year, 10 and 20 sex acts per month and 90% and 0% coverage with condom use, respectively. Although the profiles were set arbitrarily, they reflect behavioural patterns observed in the TAK cohort (data according to a standard questionnaire completed by patients prior an HIV test). Over two thirds (75%) of patients reported heterosexual contacts. The average number of casual partners per year was reported by 76% of the patients as fewer than five, six to 20 by 17% and more than 20 by 7%. At the same time, 88% reported not being in stable relationship and only 57% of patients reported using condoms.

### Utilities

Baseline utility values used for modelled patients were taken from Golicki et al. reporting utility values of general population in Poland [21]. Utility values were dependent on sex and age. When the person’s health state was changed, the baseline utility was corrected by health state utility multiplier derived from meta-analysis of utility estimates for HIV/AIDS based on time trade off method by Tengs et al. The value of utility of health status used in the Markov was 0.935 for asymptomatic HIV, 0.818 for symptomatic HIV and 0.702 for AIDS [22].

### QALY lost and additional costs of new HIV infections

The costs of new HIV infections were added in each cohort to the costs generated in the Markov model. We assumed, that treatment costs of newly infected patients were equal to the total costs estimated in the modelled cohort, discounted to end of the time horizon for the transmission model.

Additionally, we estimated life years gained per HIV-negative patients at the same age as in the input cohort, based on Polish life tables. The obtained estimate was used to calculate life years lost for every newly infected person, as the difference between life years gained (LYG) calculated for an HIV-negative patient and LYG obtained in the Markov model for the corresponding group. All life-years related calculations were discounted.

A similar assumption was adopted to estimate QALY lost. First, we calculated QALY gained per an average HIV-negative person based on Polish lifetable and utility of adult Polish population [21]. Next, we compared the estimated value and the value of QALY calculated with the Markov model, obtaining potential QALY lose by a newly infected person.

### Mortality data

General population mortality was estimated on the basis of Polish life tables and baseline characteristics of cohorts (age, gender). Mortality of HIV infected patients without any comorbidities in IIG was retrieved from Stern et al. reporting the probability of death depending on CD4+ count at cART start [23]. We fit a parametric model (exponential distribution) to retrieved data and compared estimated hazards to data and life tables published by authors (with inclusion of patients with similar characteristics to those included in Stern 2009). The estimated hazard ratio for mortality was used to modify a survival life table used in our model.

For the initial time period of DIG individuals in which they did not received cART treatment, we used a survival curve estimated in the same way as for IIG individuals, based on data reported by Kovarii et al. [24]. Then, at the start of cART treatment, survival curves for DIG patients were switched to survival curves dependent on published hazard ratios (HR) and are summarized in Table 5.

**Table 5:** Summary of estimated mortality hazard ratio used in analysis.

Category	Exponential	
	HR	95% CI
**No treatment cART^a^**	8.69	1.33–16.04
**CD4+ cells count at cART initiation^b^**		
**451–550**	2.26	1.51–3.00
**351–450 (baseline strata for CD4+ count)**	2.46	1.65–3.27
**251–350**	3.32	2.22–4.41
**151–250**	4.42	2.96–5.87
**51–150**	6.28	4.21–8.35
**0–50**	11.93	7.99–15.86

a) Based on survival curve reported in Kovari 2015

b) Based on survival curves reported in Stern 2009

**Fig 2:**
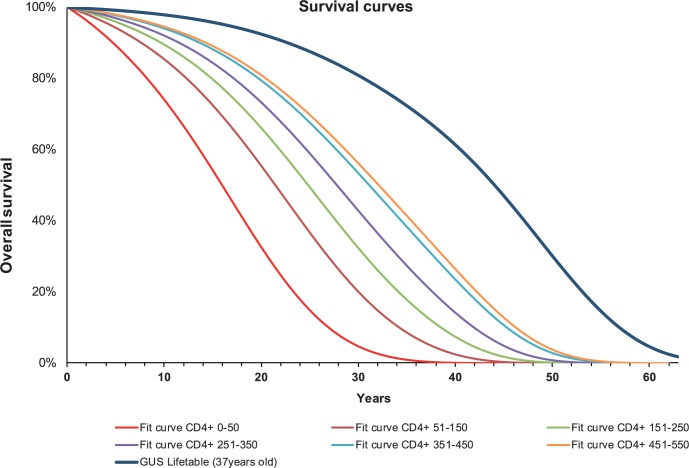
Survival curves used in base case scenario of analysis.

For the health states defined by AIDS-defining illnesses, basic mortality estimated on the basis of life tables and HR from Stern/Kovarii, was additionally increased by HR values reported by Mocroft et al. [25]. For health states defined by non-AIDS defining illnesses (Hodgkin lymphoma, lung, anal and liver cancer), we used survival curves from Hleyhel 2015, which were extrapolated by fitting exponential distribution [26]. Despite the fact that pancreatitis in patients from general population increases their mortality rate, we did not find any published data regarding mortality of patients with HIV and pancreatitis, so we made the assumption of no increase in mortality rate for these patients. Nevertheless, the probability of pancreatitis is the same regardless of the type of cART used. The above simplified assumption was referred to as conservative approach (we charged more costs in the DIG than in the IIG). In case of a fatal cardiovascular event, we calculated the probability of death, based on the data reported by Frisch-Moller et al. [17]. Fig 2 presents survival curves used for different lymphocyte CD4+ strata in the base case scenario of our analysis and compares it to lifetable for a 37 years old person from general Polish population.

### Costs

In our analysis we did not itemize the costs of specific drugs used in AIDS treatment. Instead, we assumed constant monthly costs of antiretroviral drugs based on the data reported by the National Health Fund (NHF)–the Polish public health care payer. The costs of diseases treatment included in the model were estimated on the basis of published data [27–29]. The costs of treatment of AIDS-defining illnesses were estimated on the basis of the data from the NHF relevant to average costs of hospitalization for specific indications (in Polish payment described by Diagnosis -Related Groups). We made the assumption that the treatment of severe, moderate and mild AIDS defining illnesses required 5, 3 and 1 hospitalization, respectively. The definition of AIDS defining illnesses as severe, moderate or mild was based on the study Mocroft 2009 [25]. In case of non-AIDS defining illnesses, treatments for which costs data were not available and for cardiovascular events, the costs were assumed to be equal to the cost of 1 hospitalization. Moreover, for each disease all treatment costs were charged only once (at the disease onset). The summary of cost data is presented in Table 6.

**Table 6:** Summary of cost categories adopted in the analysis.

Disease or event	Cost of treatment	Source
**Asymptomatic HIV**		
**ART Treatment**	3 218.07 PLN^a^	KAOS, AOS
**AIDS defining Illnesses**		
**Non-Hodgkin’s lymphoma**	122 683.40 PLN	DRGs^b^
**Progressive multifocal leukoencephalopathy**	32 697.35 PLN	DRGs
**Cryptococcosis**	19 618.41 PLN	DRGs
**Cerebral toxoplasmosis**	19 618.41 PLN	DRGs
**Invasive cervical carcinoma**	19 618.41 PLN	DRGs
**Bacterial pneumonia**	19 618.41 PLN	DRGs
**AIDS dementia complex**	19 618.41 PLN	DRGs
**Disseminated Mycobacterium avium disease**	19 618.41 PLN	DRGs
**Mild**	19 618.41 PLN	DRGs
**Non-AIDS defining illnesses**		
**Liver cancer**	88 216.28 PLN	DRGs
**Lung cancer**	4 005.01 PLN	NFZ 2011
**Anal cancer**	22 349.23 PLN	DRGs
**Pancreatitis**	6 573.00 PLN	DRGs
**End-stage renal disease**	66 813.75 PLN	DRGs
**Liver associated event:**	1 953.39 PLN	DRGs, Kaczor 2012
**Hodgkin's lymphoma**		
**Hodgkin's lymphoma**	4 679.28 PLN	DRGs
**Cardiovascular events and other illnesses**		
**Myocardial infarction: nonfatal**	6 101.18 PLN	DRGs
**Myocardial infarction: fatal**	6 101.18 PLN	DRGs
**Stroke: no-fatal**	7 424.15 PLN	DRGs
**Stroke: fatal**	7 424.15 PLN	DRGs
**Invasive procedures (coronary artery coronary by-pass, carotid endarterectomy)**	13 892.14 PLN	DRGs
**Fracture, inadequate trauma**	3 811.16 PLN	Amarowicz 2015
**Diabetes mellitus**	3 206.66 PLN^c^	Kinalska 2002

a) Cos per one cycle

b) DRGs—Diagnosis-Related Groups

c) Cost per one year

Costs and effects were discounted at rates of 5% and 3.5%. The cost-effectiveness threshold for incremental cost-effectiveness ratio (ICER) was set to 125 955 Polish new złoty (PLN) (29 312 EUR) according to the Ministry of Health requirements as three times a value of gross domestic product (GDP) per capita [30]

### Ethics

The TAK study obtained ethical approval from the Bioethical Committee of the Medical University of Warsaw (AKBE/99/16). All data used in the study were fully anonymized before any of the authors had accessed them.

## Results

In the base case analysis, which did not include the effect of HIV transmission, with 20-year time horizon the estimated discounted QALY of a person who started cART therapy immediately at linkage to care (IIG) was 11.29, as compared with 11.15 for a person with cART therapy delayed by 1 year. The calculated total treatment costs were 516,333 PLN for a person starting treatment immediately and 473,560 PLN for a person with one year cART delayed. The difference in QALY was therefore 0.14 and in cost per QALY reached 313,484 PLN.

In scenario with cART therapy delayed by 3 years, estimated discounted QALY was 10.35, the total treatment costs incurred were 369,129 PLN and the difference in cost per QALY gained, comparing to IIG individua,l was 156,335 PLN. In this scenario the cost-effectiveness analysis showed no financial benefit from starting cART therapy immediately, comparing to cART therapy delayed by one or three years since the linkage to care.

Next, we carried out the analysis with the risk of sexual HIV transmission (medium risk scenario) included in the model. The estimated sexual HIV transmission rate within one year of a cART therapy delay was 0.61, which is 0.59 more than for persons receiving cART immediately at linkage to care (Table 7). A higher transmission rate in persons without cART leads to additional decrease of QALY of newly infected persons and additional costs of treatment associated with new infections. Estimated additional QALY lost due to HIV transmission was 0.04 per IIG person and 1.13 in DIG and the overall difference in QALY between IIG and DI was 1.23. The additional costs of treatment associated with new infections were 277,067 PLN in DIG, that is 264,421 PLN more than for IIG persons, which leads to the total costs incurred higher by 221,648 PLN. Immediate cART therapy was found to be more cost-saving than therapy delayed by one year since the linkage to care (Table 7).

**Table 7:** Results of analysis for base case scenario (Medium Risk scenario).

Category	Results (1-year delay / 3-years delay)		
	IIG	DIG	Incremental
**Sexual HIV transmission**	0.03 / 0.08	0.61 / 1.80	-0.59 / -1.73
**Total treatment costs**	516 333 / 516 333	473 560 / 369 129	42 773 / 147 204
**Cost of treatment new infections**	12 646 / 34 208	277 067 / 577 394	-264 421 / -543 186
**Total costs (Total treatment costs + Cost of new infections)**	528 979 / 550 541	750 627 / 946 523	-221 648 / -395 982
**QALY**	11.29 / 11.29	11.15 / 10.35	0.14 / 0.94
**QALY lost**	0.04 / 0.12	1.13 / 4.43	-1.09 / -4.31
**QALY (adj)**	11.25 / 11.17	10.02 / 5.91	1.23 / 5.25
**ICER**			cost-saving / cost-saving

The estimated total sexual HIV transmission in DIG during initial three years of cART delay was 1.80, which was 1.73 more than in IIG during the same period. Estimated additional QALY lost due to HIV transmission was 0.12 in IIG and 4.43 in DIG, leading to overall difference 5.91 QALY. The additional costs of treatment associated with transmissions were 577,394 PLN in DIG, which was 543,186 PLN more than in IIG, and the total costs incurred in DIG were 395,982 PLN more than in IIG. Thus, in scenario with a three-year delay, immediate cART therapy dominates delayed cART therapy.

### Sensitivity analyses

To test, whether the model is robust and the inference is sensitive we have run the model with two additional scenarios of the transmission rate (see Methods). At low transmission risk scenario, estimated one year HIV transmission was 0.01 in IIG and 0.28 in DIG, while during three years estimated transmission was 0.04 in IIG and 0.82 in DIG. At high risk scenario, estimated transmission during one year was 0.09 in IIG and 2.07 in DIG, while during three years estimated transmission was 0.25 in IIG and 6.11 in DIG. In both, a low and high risk scenario immediate cART dominates therapy delayed by one or three years, thus, changing the risk of transmission leads to the same conclusion as in the medium risk scenario. The results of sensitivity analyses are presented in Table 8.

**Table 8:** Results of sensitive analysis (Low Risk scenario and High Risk scenario).

Category	Results (1-year delay / 3-years delay)		
	IIG	DIG	Incremental
**Low Risk Scenario**			
**Sexual HIV transmission**	0.01 / 0.04^a^	0.28 / 0.82	-0.27 / -0.78
**Total treatment costs**	516 333 / 516 333	473 560 / 369 129	42 773 / 147 204
**Cost of treatment new infections**	5 895 / 15 947	125 975 / 262 526	-120 080 / -246 580
**Total costs (Total treatment costs + Cost of new infections)**	522 228 / 532 280	599 536 / 631 656	-77 307 / -99 376
**QALY**	11.29 / 11.29	11,15 / 10.35	0.14 / 0.94
**QALY lost**	0.02 / 0.06	0.52 / 2.02	-0.50 / -1.96
**QALY (adj)**	11.27 / 11.23	10.64 / 8.33	0.63 / 2.90
**ICER**			cost-saving / cost-saving
**High Risk Scenario**			
**Sexual HIV transmission**	0.09 / 0.25	2.07 / 6.11	-1.99 / -5.86
**Total treatment costs**	516 333 / 516 333	473 560 / 369 129	42 773 / 147 204
**Cost of treatment new infections**	42 181 / 114 103	939 075 / 1 956 986	-896 894 / -1 842 882
**Total costs (Total treatment costs + Cost of new infections)**	558 515 / 630 437	1 412 636 / 2 326 115	-854 121 / -1 695 678
**QALY**	11.29 / 11.29	11.15 / 10.35	0.14 / 0.94
**QALY lost**	0.15 / 0.41	3.84 / 15.03	-3.70 / -14.62
**QALY (adj)**	11.14 / 10.88	7.31 / -4.68	3.83 / 15.56
**ICER**			cost-saving / cost-saving

a) The difference between 1-year delay / 3-years delay in IIG due to the fact that the period for which we count the number of transmission depends on the delayed therapy

## Discussion

Our analyses were performed to compare two hypothetical, independent cohorts of patients, one linked to immediate care and cART treatment, while the second cohort started cART treatment after one- or three-year delay. In base case analysis we did not include the risk of transmission while the person is not on cART, nor the costs related to new infections resulted from delaying treatment. In this case all ICER values comparing immediate cART with one year delayed cART were above the willingness-to-pay threshold. When comparing immediate cART with a three-year cART delay, 19,7% of values were cost-effective and 80,3% were above the willingness-to-pay threshold.

**Fig 3:**
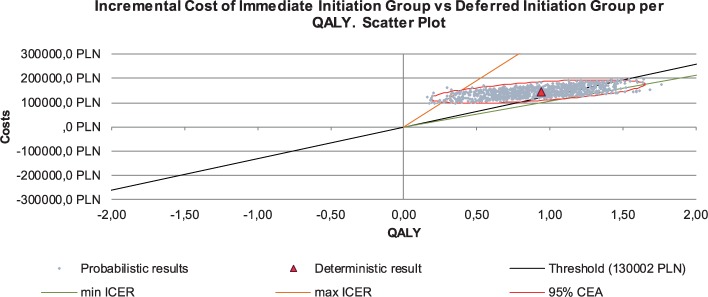
Scatter plot for PSA results.

**Fig 4:**
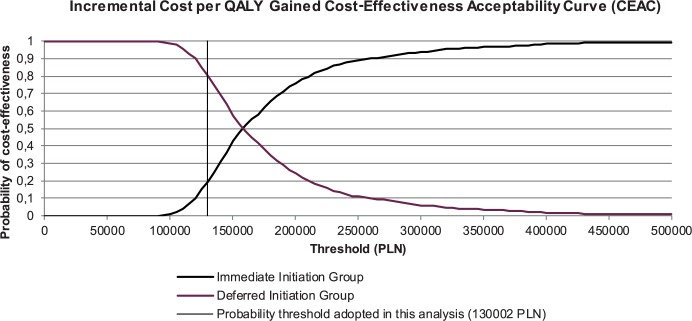
Cost-effectiveness acceptability curve for PSA simulations.

In all scenarios comprising effect of low, medium and high risk of sexual HIV transmission, all values generated with PSA showed the dominance of immediate cART therapy over both one and three years delayed cART. In all scenarios probabilistic sensitivity analyses were performed around key variables, confirming the robustness of the model (Figs 3 and 4).

This is, to our best knowledge, the first model to investigate the costs-effectiveness of cART in a central European country, which includes a benefit of protecting the HIV transmission. In many Central European countries the epidemic is now driven by sexual contacts, especially among men who have sex with men [31–35]. At the same time these countries often cannot afford to follow the most recent standards of care, namely starting treatment at the time of HIV diagnosis [36, 37]. Our work directly addresses the current needs of governments and stakeholders to base public spending on evidence-based data.

There is a number of limitations which need to be considered while interpreting our data. First of all, comprehensive evaluation of additional costs associated with new infections is relatively hard. A newly infected person can infect others, thus, during his lifetime costs associated with new, consecutive HIV infections can grow exponentially. To overcome this problem, in our analysis we included only those new infections that directly come from the modelled patient during time lag to cART therapy initiation. Moreover, the estimation of the number of newly infected persons was made only for sexual transmission, nevertheless, other ways of HIV transmission exist. It should be also emphasised, that an estimation of the sexual transmission is a very complicated process, encumbered with many uncertainties. Lack of adequate data about the number of regular or casual sexual partners, the number of sexual intercourses and the frequency of condom use can affect the number of HIV transmissions resulting in overestimation of associated costs. However, it is somewhat unrealistic to expect a high precision in information related to sexual behaviours, since persons with many sexual partners and intercourses might have a problem in recalling their sexual practices. This becomes even more difficult when psychoactive substances are used along with sexual behaviours (chemsex) [38]. It is also important to point out that we have estimated number of new HIV infections only for a period of time when patients were not on cART (maximum time 3 years) and assumed no infection occurs immediately after starting cART.

The characteristics of patients are based on relatively a small sample of 141 patients from Test and Keep in Care project. However, this is so far the only cohort providing both pre- and in-care data in Poland with information available on sexual risks and behaviours provided by persons at the time of HIV anonymous testing and linking this information with their later clinical evaluations in an HIV clinic [9, 10].

Initially the number of AIDS incidents was estimated on the basis of CD4+ T-cell counts, viremia and CD4+/CD8+ ratio, which can lead to additional uncertainty of the estimated value due to scarcity of data regarding viremia and CD4+/CD8+ ratio. Finally, we used only CD4+ T-cell counts for the estimation of the number of AIDS incidents, although such an approach still might be imprecise. Nevertheless, the assumptions made in our model are consistent with those adopted by other authors [39–41]. However, such models should be continuously updated with the most recently published better quality data, particularly on linkage to the CD4+ T-cell counts in the long-term horizon.

We have built a model including numerous epidemiological and clinical factors and based on a real-life cohort of HIV positive persons from Poland. We have shown that taking into account HIV transmission in the cost–effectiveness analyses proved immediate initiation of cART at linkage to care to be a cost-saving decision from the point of view of the Ministry of Health and national health funds in Poland. We believe these analyses are also relevant for other central European countries with comparable epidemiology and costs of HIV care.

## Supporting information

S1 File (XLS)
